# The Applications of Electrochemical Immunosensors in the Detection of Disease Biomarkers: A Review

**DOI:** 10.3390/molecules28083605

**Published:** 2023-04-20

**Authors:** Huinan Chen, Jialu Zhang, Rong Huang, Dejia Wang, Dongmei Deng, Qixian Zhang, Liqiang Luo

**Affiliations:** 1 College of Sciences, Shanghai University, Shanghai 200444, China; 2 School of Medicine, Shanghai University, Shanghai 200444, China; 3 School of Materials Science and Engineering, Shanghai University, Shanghai 200436, China; 4 Shaoxing Institute of Technology, Shanghai University, Shaoxing 312000, China

**Keywords:** biomarkers, electrochemical immunosensors, nanomaterials, detection

## Abstract

Disease-related biomarkers may serve as indicators of human disease. The clinical diagnosis of diseases may largely benefit from timely and accurate detection of biomarkers, which has been the subject of extensive investigations. Due to the specificity of antibody and antigen recognition, electrochemical immunosensors can accurately detect multiple disease biomarkers, including proteins, antigens, and enzymes. This review deals with the fundamentals and types of electrochemical immunosensors. The electrochemical immunosensors are developed using three different catalysts: redox couples, typical biological enzymes, and nanomimetic enzymes. This review also focuses on the applications of those immunosensors in the detection of cancer, Alzheimer’s disease, novel coronavirus pneumonia and other diseases. Finally, the future trends in electrochemical immunosensors are addressed in terms of achieving lower detection limits, improving electrode modification capabilities and developing composite functional materials.

## 1. Introduction

Biomarkers are significant biochemical indications of physiological abilities that are used to predict the onset or progression of diseases [1]. It is possible to detect biomarkers and predict how a disease will progress [2]. Particularly, early biomarkers are crucial for the early diagnosis of diseases and are utilized for disease diagnosis and staging [3]. Low dosages, high sensitivity, quick reaction times, and the presence of fixed biochemical reactions are characteristics of biomarkers, making them difficult to identify [4,5].

Traditionally, the clinical diagnosis of cancer relies on various medical imaging techniques to detect cancerous tissue [6]. Actually, before a malignant mass forms, biomarkers linked to the early stages of cancer appear in the blood first [7,8]. Therefore, various tumor biomarkers were applied in clinical practice as a supplement to early cancer screening [9,10]. For example, squamous cell antigen is used to detect lung cancer, and prostate specific antigen (PSA) is used to detect prostate cancer [11]. Alzheimer’s disease is a typical neurological disease of the elderly and is not curable [12]. The β protein and the P53 protein in peripheral blood and cerebrospinal fluid are the biomarkers for the clinical beginning of Alzheimer’s disease [13,14]. Additionally, procalcitonin is the marker for septicemia. The parathyroid hormone (PTH) is the marker for thyroid function, and the receptor activator nuclear factor-B biomarker is the marker for rheumatoid arthritis [15,16]. There has been some research on biomarkers for the diagnosis of diseases [17].

The sensor is normally composed of a receptor, a transducer, and a detector [18]. Immunosensing techniques combine sensors with immunological responses, in which antibodies and antigens are designed to conjugate with target analytes to produce specific changes [19]. The transducer can convert and transmit physicochemical signals in response to biological changes, which are received by the detector [20]. Constructed immunosensors in this fashion transform the concentration of the biomolecule into the quantifiable signal [21,22]. Among all biosensing principles, the electrochemical technique, which is generally sensitive, affordable and requires low power consumption, should be suitable for applications [23]. The classic case is commercial blood glucose meters for glucose determination [24]. Particularly, electrochemical immunosensors use antibodies or antigens as biomolecular recognition element components placed on the electrode surface, the formation of stable immunological complexes to generate an electrical signal, and the acquisition and output of electrochemical signals to complete the target detection (proteins, bacteria, viruses, or small molecules) [25,26]. The constructed electrochemical immunosensor has the advantages of both immunosensor and electrochemical techniques. Because it is quick, sensitive, and selective, the electrical output signal of electrochemical immunosensors is employed to analyze and detect disease biomarkers [27,28].

In this review, we supply a summary of the fundamentals and types of immunosensing. The applications of electrochemical immunosensors in the detection of disease biomarkers are reviewed and discussed in detail. In addition, the future prospects of electrochemical immunosensors in clinical diagnostics are presented.

## 2. Types of Electrochemical Immunosensors

Electrochemical devices are effective tools used for delicate measurements in bio-analysis and diagnostics [29]. The immunosensor using electrochemical devices is an ideal platform in clinical diagnosis for quantitative immunoassays, combining the benefits of electrochemical processes, immune recognition reactions and biosensor devices [30].

The high specificity and selectivity resulting from antigen–antibody immune recognition allow these immunosensors to be used in complex biological matrices such as blood, plasma, or urine. The electrochemical Immunosensors have the advantages of rapid detection, reusability, accountability, elevated throughput and the possibility of label-free detection. However, disadvantages such as difficult electrode maintenance, costly protein handling and short sensor lifetime cannot be ignored [31,32].

Typically, there are two categories of electrochemical immunosensors that are based on the accurate identification of antigen and antibody: competitive immunosensors and noncompetitive immunosensors [33].

### 2.1. Competitive Electrochemical Immunosensors

**Figure 1:**
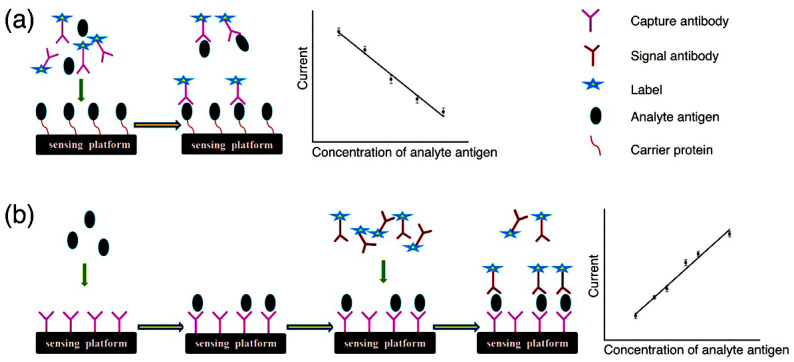
A schematic representation of (**a**) competitive immunoassays and (**b**) noncompetitive immunoassays [31]. Copyright (2016) Wiley.

Competitive immunoassays are those in which the target analyte (such as antigen, protein, and small molecule) competes recognition site on the surface of an antibody with an antigen that has been labeled with a signal molecule, as shown in Figure 1a [31]. In general, the antibody is first immobilized on the surface of the electrode and then incubated with a mixture of target analyte and quantitatively labeled antigen. Finally, the concentration of the target analyte is obtained by reading the electrical signals [34,35]. The immunoreaction signal between the antigen and the antibody is amplified into an electrical signal by the labeled signal molecule [36]. The labeled antigen competes with the target antigen for binding to the antibody, and when the concentration of the antigen in the sample is high, less of the labeled antigen will specifically bind to the antibody. Therefore, for quantitative analysis, the amount of labeled antigen interacting with the antibody is inversely proportional to the amount of antigen in the sample. The linear calibration graph is shown as a line plot in Figure 1a. The superiority of competitive immunoassays is in terms of sensitivity, selectivity, and reproducibility. However, the drawbacks of the protein’s high cost and vulnerability to inactivation have hampered its development [37,38]. A competitive electrochemical immunosensor was constructed using a competitive immunoassay.

### 2.2. Noncompetitive Electrochemical Immunosensors

Noncompetitive immunoassays involve the immunoconjugation of the chemical to be examined with two antibodies, i.e., the primary antibody and the secondary antibody, as shown in Figure 1b [33]. The primary and secondary antibodies labeled with signal molecules, which are both wrapped around the target, are combined to form an immune complex [39]. In this process, the primary antibody is immobilized on the surface of a solid matrix, which traps the antigen at the electrode interface, hence the name capture antibody. The secondary antibody binds to the antigen–antibody complex to produce a detection signal. The signal is derived from the labeled signal molecules within the system. Both the primary and secondary antibodies were overdosed, and unbound antibodies were removed by washing after each step of the reaction [40]. However, the primary and secondary antibodies can only form an immune complex in the presence of an antigen and the two antibodies do not react with each other. Because the “primary antibody–antigen–secondary antibody” structure is similarly sandwich-like shape, these immunosensors are based on noncompetitive immunoassays occasionally referred to as “sandwich type” immunosensors [41]. In this format, the labeled antibody binds specifically to the target antigen in the sample, and the concentration of the labeled antibody that labels the signal is proportional to the concentration of the antigen in the sample, a model shown in the linear calibration plot in Figure 1b.

Since noncompetitive immunosensors require primary and secondary antibodies to collectively recognize the target antigen, reducing the nonspecificity of the immune response, this approach has elevated specificity and sensitivity. However, this method is mostly suitable for the detection of biological macromolecules, since small-molecule antigens do not have adequate binding sites to bind two antibodies simultaneously [42,43]. Meanwhile, for the quantitative examination of the substance to be examined, the antibody-labeled signal molecule produces an electrical signal [44]. Noncompetitive immunosensors are impacted by a number of significant variables and challenges, including antibody surface modifications, redox probes preparation, and the regulation of antibody diffusion [45]. For these reasons, despite research demonstrating that noncompetitive immunosensors have higher sensitivity and detection limitations than competitive immunosensors, researchers heavily prefer both immunosensors [46]. There is also a kind of direct antigen–antibody binding for noncompetitive immunosensors [47]. The antibody or antigen is immobilized on the electrode and waits for the binding of the antigen or antibody to produce an electrical signal, also known as the “one-step” method [48,49].

Electrochemical immunosensors can be classified into redox couples, typical biological enzymes, and nanomimetic enzymes based on different catalytic substances. In the following sections, the catalytic substances are carried out differently, and we will present the applications in immunosensing techniques for the detection of different human disease biomarkers according to the two main categories of competitive immunosensors and noncompetitive immunosensors [50,51].

## 3. Competitive Immunosensors

Electrochemical immunoassays that have the advantages of high sensitivity, rapidity and economy are currently being investigated for application in the detection of human disease markers. Electrochemical analysis of proteins can be achieved by measuring the electrical signal output from redox-active markers in the immune complex system [52].

### 3.1. Redox-Couple-Labeled Electrochemical Immunosensors

In the presence of an electric current, redox pairs with redox qualities go through a redox reaction [53]. The redox marker is added to the electrochemical immunosensor, which produces a change in current of redox for the measurement of the target substance [54], such as ferrocene (Fc) [55], thionine [56] and Ru(bpy)_3_^2+^ [57].

**Figure 2:**
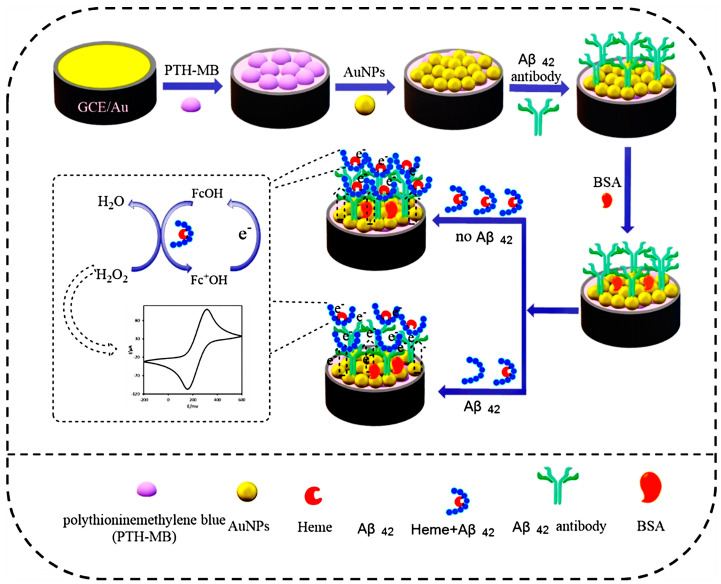
Schematic procedure for constructing the electrochemical immunosensor and determining Aβ_42_ [13]. Copyright (2021) John Wiley.

According to its redox properties and aromaticity, Fc can undergo the electrochemical redox reaction Fc − e → Fc^+^. As a signal probe, Fc is frequently used to improve electron transport [55]. Alzheimer’s disease is typically only identified after the commencement of the disease, so there is an urgent need to create diagnostic techniques for early disease markers. Ding et al. produced an immunosensor for the detection of Aβ_42_ peptides depending on the heme covalently bound to Aβ_42_ possessing peroxidase-like activity [13]. Fc was chosen as signal indicator, as seen in Figure 2. Glassy carbon electrode (GCE) was selected and sequentially modified with polysulfane-methylene blue, gold nanoparticles (AuNPs), and Aβ_42_ antibody on its surface. Aβ_42_ and heme-Aβ_42_ competitively bound to the modified electrode. Heme-Aβ_42_ complex had electrocatalytic activity, activating the redox process of H_2_O_2_ and ferrocenemethanol (FcOH). Enzymatic activity produced Fc^+^OH, which diffused to the interface of the decorated ITO surface and converted to FcOH. The competitive immunosensor has a test range of 0.056–13.7 nM and a detection limit of 25.2 pM. Using thionine as an electrical signal marker, Zhang et al. constructed a competing electrochemical immunosensor to detect the biomarker mannose on the surface of tumor cells [56]. Thionine, the nanomaterial multi-walled carbon nanotubes (MWNT), Au, and the mannose recognition protein Con A were used to create the initial version of the nanoprobe MWNT/Au/Con A. Afterwards, MWNT and thionine dimer were used to modify the GCE surface. The resulting GCE/MWNT/AuNPs/thiomannosyl dimer sensor competes with the tumor cell surface myosin for binding to the nanoribbon, and the electrical signal of the thionine is negatively correlated with the cancer cell concentration. Results from this sensor showed detection limits of 20 mL^−1^ and 35 mL^−1^ for two hepatocellular carcinoma cells, QGY-7701 and QGY-7703.

### 3.2. Enzyme-Labeled Electrochemical Immunosensors

An enzyme is a protein or RNA with biocatalytic function. Biological enzymes are widely used as markers in immunoassays due to their efficient catalytic activity, substrate specificity, and selectivity for biological reactions. An essential technique in the creation of immunosensors is the labeling of antibodies using natural biological enzymes [22]. Enzymes are capable of catalyzing the redox reactions of substrates, in which electron transfer occurs and an enhanced electrical signal is produced [58]. They have the advantage of being quick and effective. However, the intrinsic instability and susceptibility of biological enzymes to deactivation limit their catalytic effect.

#### 3.2.1. Horseradish Peroxidase

Horseradish peroxidase (HRP) is among the most frequently employed enzymes in bioanalysis [59]. The use of HRP to identify low abundances of compounds to be tested is possible due to its stability, high recovery rate, effectiveness in amplifying weak signals, and stability. However, as it is challenging to use HRP to directly mark antibodies, many researchers have labeled HRP on inorganic nanomaterials to be used [60]. In order to detect the depressive disorder marker HSP70, Sun synthesized conducting polymer polyaniline-modified graphene quantum dots (PAGD), which were employed as electrode modification materials along with target HSP70 to build a functionalized HSP70/PAGD/GCE immunosensor [61]. The HSP70 in the test analyte and the HSP70 on the electrode surface compete with each other to bind the HRP-tagged antibody. The linear range of HSP70 is 0.0976–100 ng mL^−1^, with detection limits as low as 0.05 ng mL^−1^. Freire et al. also used HRP catalysis to create an innovative immunosensor for the detection of SARS-CoV-2 nucleocapsid protein (N protein) of novel coronavirus pneumonia [62]. Graphene-SPEC capture the N protein via amide bond, followed by IgG-SARS-CoV-2 N protein, anti-IgG-HRP sequentially binds to the immunosensor, and HRP is used to catalyze the TMB substrate reaction to generate electrical signals. The constructed device has a detection range of 1:1000–1:200 *v*/*v* for SARS-CoV-2.

#### 3.2.2. Alkaline Phosphatase

**Figure 3:**
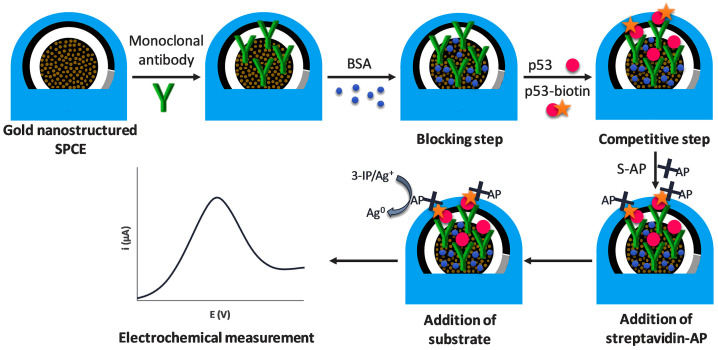
Scheme of the immunosensing strategy for the detection of unfolded p53 [14]. Copyright (2020) Elsevier.

The alkaline phosphatase (ALP) is a zinc-containing glycoprotein that hydrolyzes a variety of natural and synthetic phosphate monoester substrates. In the field of immunosensing, ALP is usually labeled with antibody and then reacted with substrate to generate electroactive products that can convert antibody–antigen-bound biological signals into electrical signals. ALP has been used to construct immunosensors for the detection of disease markers [63]. Gutierrez et al. developed an enzymatic immunosensor based on gold nanostructured carbon coating SPEC for accurate detection of the Alzheimer’s disease marker p53 protein (Figure 3) [14]. The modified immunosensor uses 3-indoxyl phosphate and silver ions as the enzymatic substrate and ALP as the labeling enzyme. The electrochemical signal in the enzyme reduction system is produced by silver ions in solution that reduce to metallic silver (Ag^0^). For blood samples, the immunosensor exhibits the low detection limit of 0.05 nM.

### 3.3. Nanomaterial-Based Electrochemical Immunosensors

Nanomaterials are frequently exploited in the field of electrochemical immunosensing because of their exceptional physical and chemical characteristics [64]. More redox couples or enzymes can be loaded onto nanomaterials with the considerable specific surface area to increase the redox reaction within the sensor system as well as to bind more antigens for analysis [65]. The catalytic activity of nanomaterials allows them to directly catalyze substrate processes. Nanoenzymes were widely developed due to their advantages of low cost, simplicity in production, and excellent stability, which may have the potential to replace traditional enzymes [66]. The disadvantages of traditional enzymes, such as high cost, poor stability and affected by the environment, also have greatly contributed to the development of artificial enzymes [67].

#### 3.3.1. Metal Nanomaterials

Nanoscale metal complexes containing nanosized grains made of one or more metals are known as metal nanomaterials. Metal nanoparticles are great for designing electrochemical immunosensors because they have good electron transport capabilities [68].

Several studies have used nanomaterials for cancer biomarker detection. A biomarker called E-selectin is strongly correlated with vascular lesions. A gold nanoparticle wrapped around CuO nanorods (Au-CuO NCs) was created by Zhao et al. and used in the field of E-selectin immunodetection [69]. The E-selectin antigen CD62E adsorbed on the Au-CuO surface to forms CD62E-Au-CuO. CD62E-Au-CuO competes with E-selectin for binding to the anti-CD62E-modified GCE. As the concentration of E-selectin increases, both the amount of CD62E-Au-CuO and the value of the generated current decrease. The final detection limit was 226 pg mL^−1^, and the detection range was 0.500–500 ng mL^−1^. Carbon nanofibers (CNF) are ideally suited for use in biosensors payable to their large specific surface area. Eissa et al. invented a new electrochemical method for the detection of SARS-CoV-2, utilizing the stepwise alteration of CNF, nucleocapsid (N) protein, and N protein antibody to functionalize SPEC electrodes [70]. Absorbent cotton padding is applied to modified electrodes as a sample collection platform to allow direct collection of nasopharyngeal samples instead of swabs. Well established detection limits (0.8 pg mL^−1^) and good sensitivity characterize the electrochemical detection results. Mucin-1 (MUC-1) is a common biomarker for breast cancer. To detect MUC-1, Rashid et al. first adapted the electrode surface using gelatin in order to modify the MUC-1 antibody, and then used MWCNT as a substrate to covalently bind dopamine to the MUC-1 antibody to form a signal probe [71]. Finally, the modified electrode, the signal probe, and the target MUC-1 form a competing immunosensor. Among them, dopamine acts as an electron donor, effectively amplifying electrical signals. The immunosensor achieves a highly sensitive detection of MUC-1, with a detection limit and range of 0.05–940 U mL^−1^ and 0.1 U mL^−1^, respectively.

#### 3.3.2. Magnetic Nanomaterials

Magnetic materials are substances that produce magnetism, including elements such as iron, cobalt, nickel and their alloys, which are easily separated from the medium in a magnetic field. Because of their high specific surface area and ease of surface modification, magnetic materials are employed in immunosensing. However, the low electrical conductivity of magnetic beads hinders charge transport. Therefore, when designing im-munosensors, magnetic beads (MBs) may undergo significant surface alteration [72].

Kalyani et al. used chitosan as a substrate for wrapping MWCNT and magnetite nanoparticles (CS-MWCNT-Fe_3_O_4_), which can be used as a signal amplification element to load large amounts of antibodies [48]. As an immunosensor for capturing potential endometriotic biomarker carbohydrate antigen 19-9, the CS-MWCNT-Fe_3_O_4_ nanocomposite is deposited on GCE and immobilizes anti-carbohydrate antigen 19-9 antibody. The detection limit is 0.163 pg mL^−1^, and the detection range is 1.0 pg mL^−1^–100 ng mL^−1^.

## 4. Noncompetitive Immunosensors

It is well known that noncompetitive immunoassays typically exhibit higher specificity, lower cross-reactivity, and a wider working range than competitive immunoassays. In this chapter, we present research advances in noncompetitive immunosensors in the field of disease marker detection.

### 4.1. Redox-Couple-Labeled Electrochemical Immunosensors

**Figure 4:**
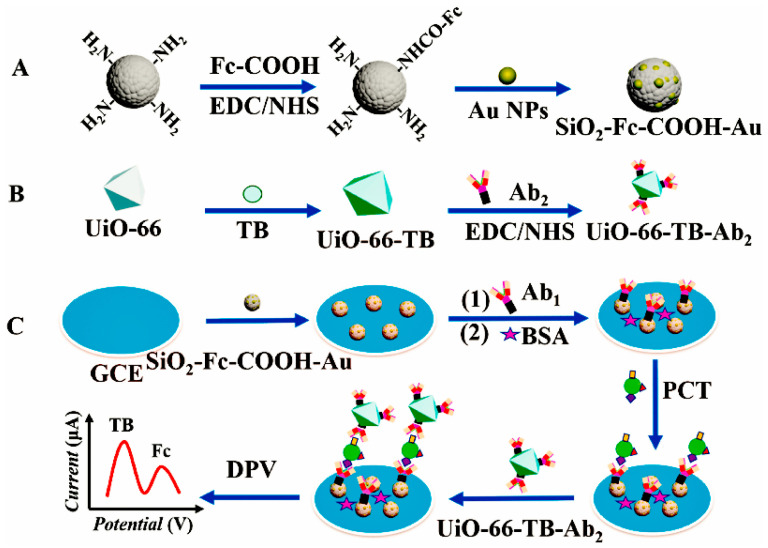
Immunosensor preparation mechanism diagram. (**A**) the preparation of SiO_2_–Fc–COOH–Au. (**B**) the preparation of UiO–66–TB–Ab_2_ (**C**) the preparation of the electrochemical immunosensor [74]. Copyright (2021) Elsevier.

Toluidine blue (TB) is a water-soluble zine redox dye that works well as electrical mediator for immunosensors [73]. Using two signaling molecules, Fc and TB, a ratiometricelectrochemical immunosensor for the procalcitonin septicemia (PCT) marker was created by Miao et al. [74]. As shown in Figure 4, the AuNP covalently bound SiO_2_-Fc-COOH nanocomposite was synthesized and selected as the matrix material for immobilizing anti-PCT antibody on GCE because Fc possesses redox characteristics and gold enhances conductivity. The TB-labeled metal–organic framework UiO-66 (UiO-66-TB) served as a signal marker owing to the immune interaction between PCT and the secondary antibody. An immunosensor of the sandwich type was constructed in the presence of target PCT. A linear relationship exists between the ratio of the dual electrical signals and PCT concentration, with an increase in PCT concentration leading to an increase in peak oxidation current of TB and a decrease in the peak oxidation signal of Fc. The linear detection range of the sandwich-type immunosensor was 1 pg mL^−1^–100 ng mL^−1^, with a detection limit of 0.3 pg mL^−1^.

### 4.2. Enzyme-Labeled Electrochemical Immunosensor

In a noncompetitive immunosensor, the enzyme-labeled antibody probe binds directly to the antigen. The antigen is sandwiched between the two antibodies, the substrate is enzymatically cleaved to produce the electroactive material, and the redox cycle of the recorded product results in an enhanced electrochemical signal. The sensitivity of the sensor is highly enhanced [75].

#### 4.2.1. Horseradish Peroxidase

There are several HRP-catalyzed immunosensors studied that are cancer-related biomarkers for detection. Gold–silver hybrid nanomaterials and graphene were added to the electrode by Nakhjavani et al. to modify it for binding with the primary antibody [76]. Sandwich immunological structures are made up of the primary antibody, carcinoembryonic antigen (CEA), and the secondary antibody that has been HRP-labeled. CEA is a broad-spectrum tumor biomarker employed in the diagnosis of several types of cancer. HQ catalyzed by HRP/H_2_O_2_ produces strong electrical impulses, which are related to the CEA. Li et al. developed an immunosensor (AuNPs/Ab1/BSA/CA–125/glucoseoxidase (GOx)/HRP@metal–organic framework (ZIF-90)-Ab2) for the detection of the ovarian cancer marker carbohydrate antigen 125 using GOx/HRP@ZIF-90 nanomaterials as signal amplification components [77]. GOx and HRP cascade amplification in the system allow for the sensitive detection of carbohydrate antigen 125 in the range of 0.1 pg mL^−1^–40 ng mL^−1^, with a detection limit of 0.05 pg mL^−1^.

**Figure 5:**
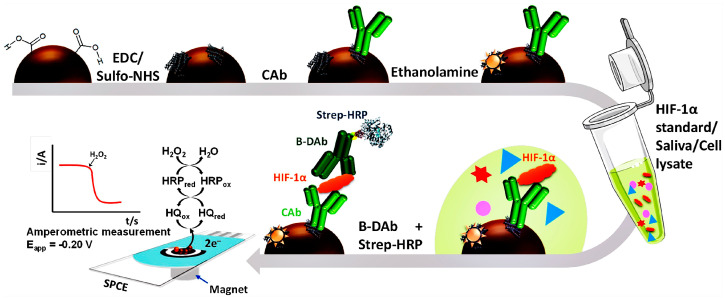
Schematic display and reactions involved in the MB–based immunoassay for the determination of HIF–1α [80]. Copyright (2020) Elsevier.

In addition to cancer, HRP is also used to construct other disease-related markers. Feng et al. used a synthetic covalent organic backbone loaded with HRP as a signal probe to construct an immunosensor that catalyzes the conversion of hydroquinone (HQ) to benzoquinone (BQ) by the synergistic action of HRP and hydrogen peroxide to generate an electrochemical signal [78]. The sensor had a detection limit of 1.7 pg mL^−1^ and was capable of detecting cardiac troponin I (cTnI) over a linear range of 5 pg mL^−1^–10 ng mL^−1^. In order to detect rheumarthritis marker receptor activator nuclear factor-B using an electrochemical approach, Valverde et al. first developed an HRP-catalyzed sandwich immunosensor (HQ) using the redox of 4-aminobenzoic acid (p-ABA) substrates catalyzed by H_2_O_2_/hydroquinone [79]. The detection range is 10.4–1000 pg mL^−1^, and the detection limit is 3.1 pg mL^−1^. As seen in Figure 5, the first MB-based immunosensor for the detection of hypoxia-inducible factor-1 alpha (HIF–1α) was created by Martin et al., by immunoconjugating MBs with HRP-modified antibodies and using HRP/H_2_O_2_ to catalyze HQ to produce an electrochemical signal (with a detection limit as low as 76 pg mL^−1^) [80].

Eventually, HRP can also be applied to virus detection. Vasquez et al. created an enzyme-amplified electrochemical immunosensor for the detection of SARS-CoV-2 spike protein [81]. The immunosensing device retains the target spike protein in a sandwich between anti-spike antibody modified on MBs and a biotin-anti-ACE2 antibody, biotinylated antibody conjugated to HRP-labeled streptavidin. In the presence of H_2_O_2_, HRP catalyzes the conversion of TMB substrates into an electrochemical signal. The immunosensor has a detection range of 1–5 × 10^5^ copies mL^−1^ and a detection limit of 22.5 ng mL^−1^.

#### 4.2.2. Alkaline Phosphatase

**Figure 6:**
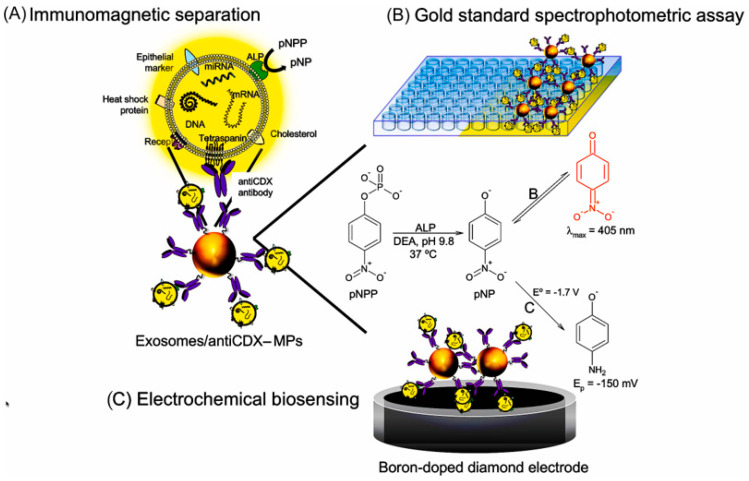
Different approaches for the detection of alkaline phosphatase (ALP) activity in osteoblast–derived exosomes by optical readout and electrochemical biosensor [28]. Copyright (2022) Elsevier.

ALP has been used to detect a variety of disease markers, such as exosomes, proteins, and antigens. Exosome biomarkers for diagnostic or prognostic reasons appear to be fast translating into applications [82,83]. Moura et al. first used ALP-labeled magnetic beads, as depicted in Figure 6 [28]. The difference between normal samples and samples from breast cancer patients is determined by concurrently detecting two proteins on breast cancer exosomes. The detection limit is 4.39 mU L^−1^, representing to 10^5^ exosomes μL^−1^. Lee et al. developed a sandwich immunosensor to measure extracellular vesicles (EV) [84]. The anti-EGFR primary antibodies were immobilized onto magnetic bead-coated AuNP-modified ITO electrodes. The anti-CD63 secondary antibodies were cross-linked with ALP, which can catalyze the production of L-ascorbic acid (AA) from the substrate ascorbyl-2-phosphate (AAP). Analytical indications that were quantitatively correlated to the concentration of EV were the redox peak current of ALP. The biosensor platform has an EV detection limit of 10^8^ particles mL^−1^ for glioblastoma multiforme.

Kim et al. constructed an ALP-catalyzed electrochemical immunosensor to successfully detect IgG in human serum by catalyzing the redox of *p*-APP substrates [85]. The detection limit and range are 1 IU mL^−1^ and 3–30 IU mL^−1^, respectively. Cao et al. developed ALP-IgG-labeled GNPs/Ab2-based electrochemical microfluidic devices for the sensitive detection of human chorionic gonadotropin via the redox of para-nitrophenyl phosphate (p-NPP) substrates hydrolyzed to p-nitrophenol (p-NP) [86]. The detection limit is 0.36 mIU L^−1^, with a detection range of 1.0–100 mIU L^−1^.

#### 4.2.3. Urease

**Figure 7:**
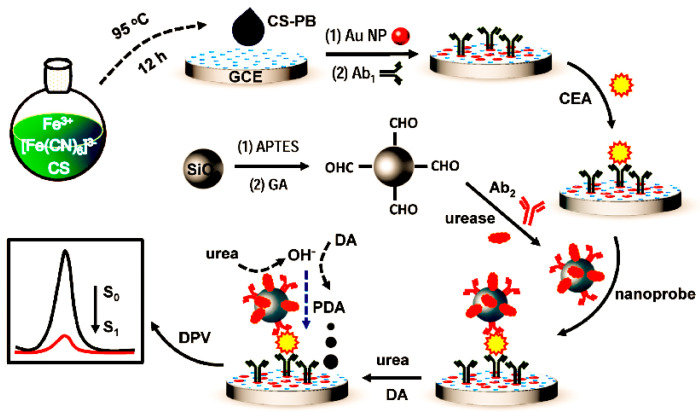
Schematic illustration of the preparation process of the CS–PB–based immunosensor and its electrochemical signal transduction principle [87]. Copyright (2022) Elsevier.

Urease, the first enzyme to crystallize and be confirmed as a protein, catalyzes the breakdown of the urea substrate, releasing large amounts of hydroxide ions. Huang et al. utilized the catalytic action of urease to detect CEA as shown in Figure 7 [87]. The electrochemical indicator chitosan-prussian blue (CS-PB) nanocomposite and primary antibody are modified on the GCE. The urease and secondary antibody modified to silica nanoparticle serve as immune nanoprobe. The modified GCE, urease-functionalized silica nanoprobe, and CEA constitute a sandwich immunosensor. In this system, urease catalyzes the conversion of dopamine into polydopamine (DPA), which releases hydroxyl radicals and increases the inhibition of the PB electrochemical signal. The inhibition of electrical signal comes mainly from urease-catalyzed PDA deposition and multi-enzyme signal amplification of silica nanoprobe. The results indicate a detection limit of 0.042 pg mL^−1^ for CEA, with a detection range from 0.1 pg mL^−1^ to 100 ng mL^−1^. In this work, two different catalytic substances are used—the biological enzyme urease and nanomaterials—which together increase their inhibitory effect on electrical signals and thus increase the sensitivity of the immunosensors.

### 4.3. Nanomaterial-Based Electrochemical Immunosensors

The advantages of low cost, high stability and the unique physicochemical properties of nanomaterials have led to their reuse in electrochemical immunoassays [88].

#### 4.3.1. Metal Nanomaterials

**Figure 8:**
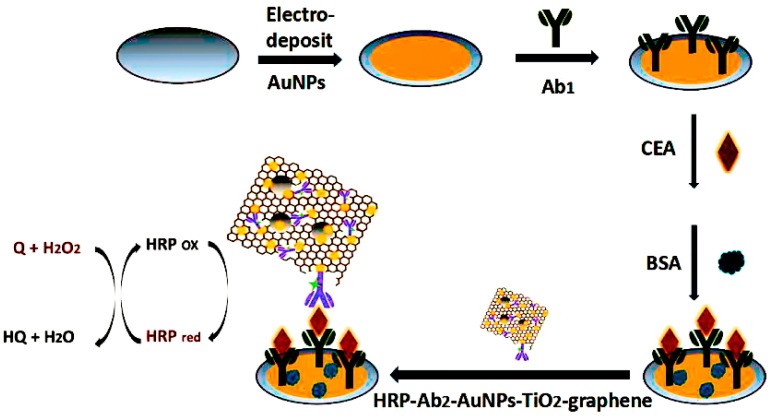
Schematic diagram of a CEA electrochemical immunosensor [89]. Copyright (2018) Elsevier.

In order to create a metallic nanomaterial with good electron transport properties, Chen et al. combined graphene, Au and metallic titanium dioxide (TiO_2)_ [89]. An HRP-labeled secondary antibody was coupled with the graphene metal nanocomposite nanocomplex to create an immunocomplex that was catalytic, as shown in Figure 8. To identify CEA, metal- and enzyme-modified graphene nanocomplexes were added to the primary antibody-labeled electrode’s surface. This immunosensor had a 3.33 pg mL^−1^ detection limit for CEA. Silver@cerium oxide-gold (Ag@CeO_2_-Au) nanocomposites were made by Chen et al. [90]. Because the metal Ce has a good capacity for oxidation and reduction, this ability is enhanced when Ce and Ag function simultaneously. Therefore, an immunosensor made of Ag@CeO_2_-Au is sensitive enough to detect CEA.

**Figure 9:**
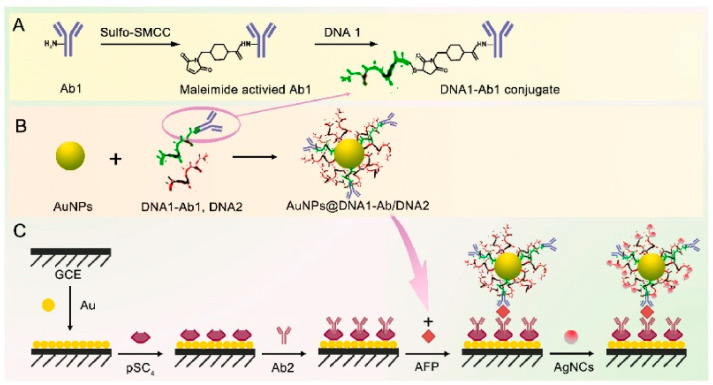
Schematic illustration of the preparation procedure for the sandwich-type electrochemical immunosensor. (**A**) The DNA1-Ab1 conjugate. (**B**) The formation of AuNPs@DNA1-Ab1/DNA2. (**C**) The principle of the proposed immunesensor [93]. Copyright (2023) Elsevier.

Alpha-fetoprotein (AFP) is among the earlier cancer biomarkers examined in humans and is a key biomarker for the early detection of hepatocellular carcinoma [91]. Alpha-fetoprotein detection aids in the early diagnosis and treatment of hepatocellular cancer [92]. Regarding the goal of detecting AFP, Chen et al. created novel spherical nucleic acid-linked silver nanocluster (AuNPs@DNA-AgNCs) nanocomplexes, as depicted in Figure 9 [93]. Two distinct DNA were attached to AuNPs, one of which was connected to an anti-AFP secondary antibody and the other of which acted as a template for the electrical signaling molecule AgNCs. The surface of GCE was successively treated with Au film and p-sulfonated calix [4] arene (pSC_4_) to increase the electrode interface’s electrical conductivity and create an environment favorable for antibody attachment. The electrochemical immunosensor was formed of AuNPs@DNA-AgNCs nanocomplexes, AFP, and Ab1/pSC_4_/Au/GCE. The relationship between the electrical signal of Ag^+^ and AFP concentration is linear. The AFP detection range is exceptionally broad (0.001–100 ng mL^−1^) and has a knockdown detection limit (7.74 fg mL^−1^).

**Figure 10:**
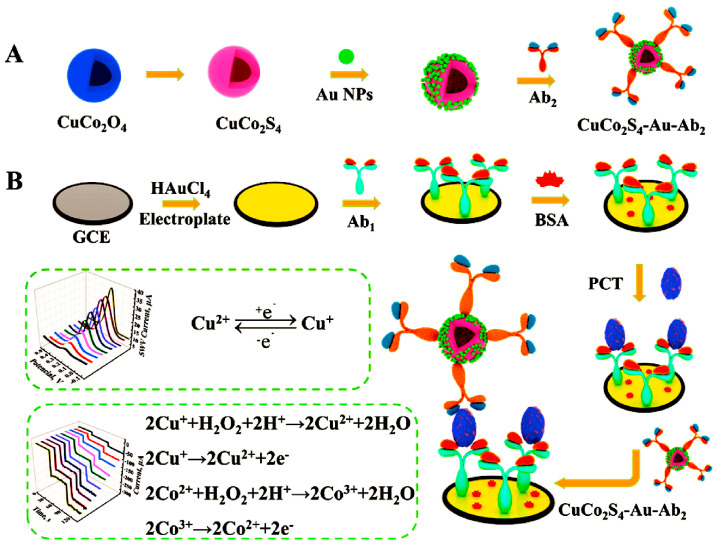
Schematic diagram of the preparation of the nanometal immunosensor complex CuCo_2_S_4_–Au–Ab_2_ (**A**) and the construction of the immunosensor (**B**) [94]. Copyright (2020) Elsevier.

When two or more metals are combined to generate metal nanostructures, there are positive metal–metal synergistic effects. Li et al. synthesized the bimetallic compound CuCo_2_S_4_ to construct a two-signal sandwich immunosensor for the detection of procalcitonin (PCT) [94]. As shown in Figure 10, the reduction reaction of Cu^2+^ and the catalytic reaction of Co^2+^ produced the dual signal. The signal accuracy and sensitivity were enhanced by the dual signal bimetal, resulting in an excellent down to fg level detection limit for PCT (82.6 fg mL^−1^). 

Medetalibeyoglu et al. used the MXene@AuNP signal-amplifying probe, AuNPs, and the p-aminothiophenol-functionalized graphene oxide co-modified GCE immunosensor platform to immunobind a sandwich electrochemical immunosensor for the detection of PSA [95]. The immunosensor provided excellent detection of PSA, and its detection limit was determined to be 3.0 fg mL^−1^ and the linearity range to be 0.01–1.0 pg mL^−1^, respectively. Prasad et al. developed an immunoassay employing a single component of GO paper-based electrode nanocomposites and AuNP-modified anti-PEAK1, in which the synthesis of AuNP-modified anti-PEAK1 was utilized to electrocatalytically reduce PEAK1 to deliver electrical pulses by redox reaction of potassium ferricyanide for sensitive pancreatic cancer biomarker PEAK1 detection [96]. The final detection limit is 10 pg mL^−1^, with a detection range of 10–10^6^ pg mL^−1^.

**Figure 11:**
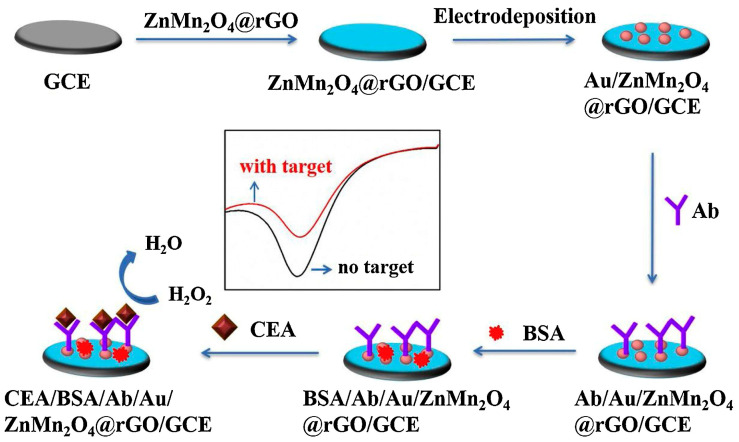
The schematic diagram of the amperometric immunosensor preparation [98]. Copyright (2021) Elsevier.

Direct antigen–antibody binding for noncompetitive immunosensors has also been achieved for the detection of biomarkers [97]. Fan et al. used a hydrothermal method to create an innovative ZnMn_2_O_4_@reduced graphene oxide composite, in which the spinel structure of ZnMn_2_O_4_ had a significant electrocatalytic impact on H_2_O_2_ reduction and reduced graphene oxide might enhance conductivity, as depicted in Figure 11 [98]. Construction of immunosensors was achieved by dropwise addition of the ZnMn_2_O_4_@reduced graphene oxide composite to GCE. The striking variation in the electrocatalytic current of ZnMn_2_O_4_@reduced graphene oxide to H_2_O_2_ demonstrates an immunoreaction between CEA antibody and CEA. The linear range of the immunosensor is 0.01–50 ng mL^−1^, with the detection limit of 1.93 pg mL^−1^.

**Figure 12:**
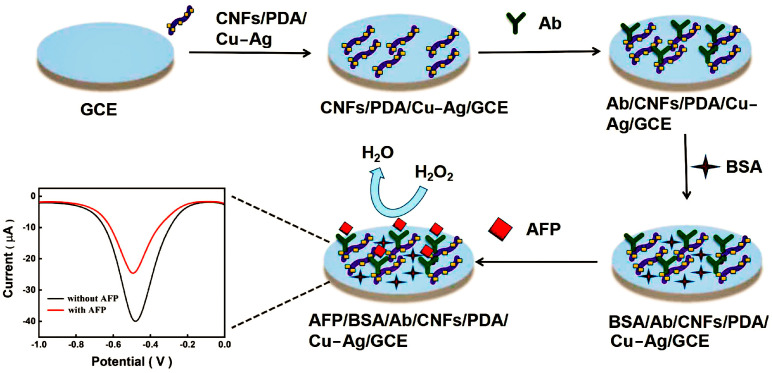
Schematic illustration of the electrochemical immunosensor preparation [99]. Copyright (2022) Elsevier.

Liu et al. built a competitive electrochemical immunosensor for the detection of AFP combining cellulose nanofibrils (CNF)/DPA/Cu-Ag nanocomposite as nanoenzymes, as seen in Figure 12 [99]. By functionalizing CNF with Ag and Cu nanoparticles on DPA using the electroless deposition method, a CNF/DPA/Cu-Ag nanocomposite was created. The AFP ultrasensitive quantitative analysis was achieved by amplifying the electrical analysis signal of the immunoreaction at the interface between the AFP and the anti-AFP antibody using the produced nanocomposite. The created immunosensor has an expansive linear range (0.01–100 ng mL^−1^) and a small detection limit (4.27 pg mL^−1^). In summary, there are two major pathways for metal nanomaterial application in electrochemical immunosensors: one is that nanomaterial modifications at the working electrodes facilitate electron transfer between the electrodes and solution transfer, while allowing the adsorption of a large number of trapped probes for stronger signals; the other is that the capture probe is first immobilized on the electrode, and then metal nanomaterial-modified probes are added to construct the sandwich structure. The signal strength is then amplified by exploiting the catalytic of the probe and nanomaterial surfaces.

#### 4.3.2. Magnetic Nanomaterials

**Figure 13:**
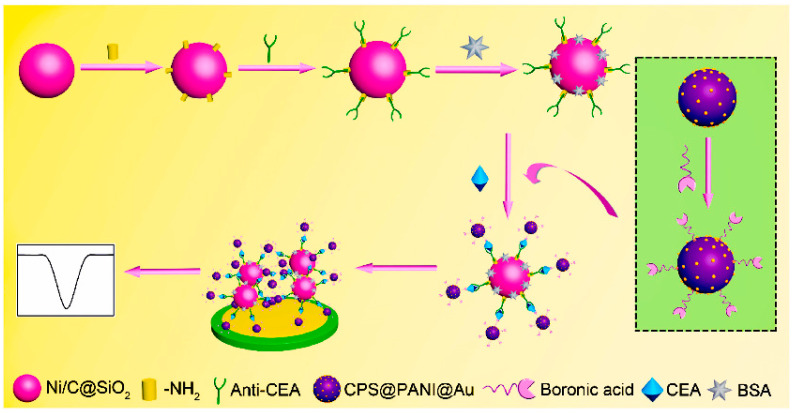
Diagrammatic representation of the steps involved in creating a sandwich–style electrochemical biosensor [100]. Copyright (2021) Elsevier.

Magnetic nanomaterials have the advantages of temperature stability, low power consumption and small size in the preparation of noncompetitive immunosensors, which improve the sensitivity of detection. Song et al. exploited hollow silica nanospheres with a magnetic outer coating of nickel/carbon as the immunological platform and carboxymethylated polystyrene@polyaniline@gold functionalized by boric acid as the electrical signal probe to build an immunosensor to detect CEA, as seen in Figure 13 [100]. The detection limit of CEA is 1.56 pg mL^−1^, and the detection range is 0.006 pg mL^−1^–12 ng mL^−1^.

**Figure 14:**
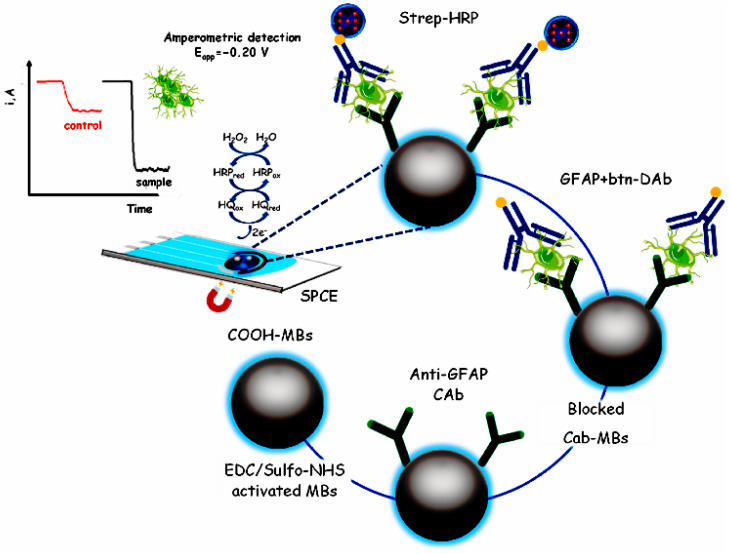
Schematic display of the MB–assisted immunoplatform preparation for the amperometric assay of GFAP [101]. Copyright (2022) Elsevier.

Bolukbasi et al. devised a Ab1/Fe_3_O_4_ NPs@ covalent organic framework (COF)/AuNPs immobilized at the GCE immunosensor platform Ab2/MNPs@SiO_2_@TiO_2_ signal enhancing probes to fabricate a sandwich immunosensor to detect AFP [72]. The fabricated immunosensor has a linear range of 0.01–1.00 pg mL^−1^ and a limit of detection of 3.30 fg mL^−1^. Several antibody-coated immunomagnetic beads (IBM) were employed by Moura et al. to identify particular tetraspanin receptors on the surface of exosomes for the detection of breast cancer exosomes [28]. The detection threshold of electrochemical sensor for exosomes was 10^5^ μL^−1^. The Alzheimer’s disease marker glial fibrillary acidic protein (GFAP) was first discovered by Ozcelikay et al. using IMB and HRP (Figure 14) [101]. The IMB served as a target enrichment, and the concentration of GFAP was proportional to the electrical signal produced by the catalysis of H_2_O_2_ by HRP. The developed immunoplatform effectively detects endogenous GFAP from the patient brain (detection limit is 67 pg mL^−1^). A sandwich immunocomplex was created by Fabiani when the anti-IgG-coated IMB interacted with the SARS-CoV-2 surface nucleocapsid protein, the secondary antibody anti-IgG-tagged ALP, and SARS-CoV-2 [102]. The magnetic isolation of the magnetic beads associated with SARS-CoV-2 in solution on SPEC, which is catalyzed by AP to generate electrochemical signal, was accomplished by magnetization. The immunoassay has a detection limit of 8 ng mL^−1^ in saliva and enables quick detection of SARS-CoV-2 within 30 min. MBs were chosen by Durmus et al. as carriers to create an electrochemical immunosensor for the sensitive and precise measurement of SARS-CoV-2 virus and its variations in nasopharyngeal swabs [103]. The SARS-CoV-2 virus binding to Ab-tagged MB is easily isolated from and enriched by magnetic action. The final detection thresholds for SARS-CoV-2 virus and its variants were 0.93 ng mL^−1^ and 0.99 ng mL^−1^, respectively.

Numerous immunosensors were developed for the detection of various diseases, such as cancer, Alzheimer’s disease and novel coronavirus pneumonia. It is clear from the examples listed in the text that the type of immunosensor is related to the size of the target analyte. Noncompetitive immunosensors have a higher sensitivity and are better suited for biomolecular detection since it requires two antibodies to bind to the target. Small-molecule targets are better suited for detection with competing immunosensors. Both types of competitive and noncompetitive immunosensors have undergone substantial research and one cannot exist without the other.

## 5. Conclusions

Nowadays, various diseases are serious threats to human life, thus early diagnosis of diseases through biomarkers is particularly critical. Electrochemical immuosensors play an essential role in biomarker detection. In this review, we highlight the developments of electrochemical immunosensors in the diagnosis of various disease markers in the last five years. Immunosensors are classified by type into two main classes: competitive and noncompetitive, each with three different catalysts: redox couples, typical biological enzymes, and nanomimetic enzymes. This is complicated by the low levels of disease biomarkers and the short detection times required for their detection. In contrast, electrochemical immuonsensors transform the biological signal of antigen–antibody binding into an electrical signal during the reaction process, combining the high sensitivity, rapid signal response, ability to detect low-abundance samples, ease of use and low cost of electrochemical and immunosensing with outstanding advantages in the detection of biomarkers.

Depending on the combination of principle types, competitive methods amplify immunoreactive signals through labeled molecules to obtain electrical signals with high sensitivity and great selectivity, and are suitable for the detection of small molecules or semantigens. Noncompetitive immunosensors are highly sensitive and have low nonspecific adsorption because they use two antibodies to bind the same antigen and are suitable for the detection of biological macromolecules. Direct antigen–antibody binding for noncompetitive immunosensors has the advantage of simplicity and low cost, but the method requires attention to the problem of background interference. Three main classes of catalytic substances, redox small molecules, enzymes and nanomaterials, can convert immunoreactive signals into electrical signals and amplify them, depending on the type of immunosensor.

A redox couple is labeled on antigens, antibodies or materials to generate electrical signals. Because of their own catalytic action, biological enzymes are used to catalyze characteristic substrates to generate electrical signals. Due to the costly and difficult to preserve nature of biological enzymes, a variety of nanomimetic enzymes were designed and studied for catalyzing the generation of electrical signals in the construction of sensors.

Immunosensors allow for sensitive and rapid detection of biomarkers. However, immunosensors also have drawbacks, such as invalidity at excessive concentrations, a short lifetime, and limitations by electrochemical detection equipment. The following are the key approaches for future development of immunosensors: 1. The goal is to achieve fmol or even lower detection limits in order to shorten analysis times and increase analytical sensitivity. 2. The requirement to create strategies for passivating electrode surfaces to thwart nonspecific adsorption of biological materials at the electrode interface. 3. The creation of composite functional materials, especially when combined with numerous amplification techniques, enhances the analytical capabilities of electrochemical enhancers to enhance the analytical performance of electrochemical immunosensors. In conclusion, we believe that electrochemical sensors have a bright future in disease biomarker detection. Building on existing research, the establishment of original methods for joint detection in the fields of immunology, novel nanomaterials, and electrochemistry will continue to advance research on early diagnosis of diseases, and point-of-care testing for diseases will be realized at an early date.

## Data Availability

Not applicable.
